# An efficient algorithm for systematic analysis of nucleotide strings suitable for siRNA design

**DOI:** 10.1186/1756-0500-4-168

**Published:** 2011-05-27

**Authors:** Ancha Baranova, Jonathan Bode, Ganiraju Manyam, Maria Emelianenko

**Affiliations:** 1 School of Systems Biology, George Mason University, Fairfax VA, USA; 2 Research Center for Medical Genetics, RAMS, Moskvorechie Str., 1, Moscow, Russian Federation; 3 Department of Mathematical Sciences, George Mason University, Fairfax VA 22030, USA; 4 Bioinformatics & Computational Biology Dept, The UT MD Anderson Cancer Center, Houston, TX, USA

## Abstract

**Background:**

The "off-target" silencing effect hinders the development of siRNA-based therapeutic and research applications. Existing solutions for finding possible locations of siRNA seats within a large database of genes are either too slow, miss a portion of the targets, or are simply not designed to handle a very large number of queries. We propose a new approach that reduces the computational time as compared to existing techniques.

**Findings:**

The proposed method employs tree-based storage in a form of a modified truncated suffix tree to sort all possible short string substrings within given set of strings (i.e. transcriptome). Using the new algorithm, we pre-computed a list of the best siRNA locations within each human gene ("siRNA seats"). siRNAs designed to reside within siRNA seats are less likely to hybridize off-target. These siRNA seats could be used as an input for the traditional "set-of-rules" type of siRNA designing software. The list of siRNA seats is available through a publicly available database located at http://web.cos.gmu.edu/~gmanyam/siRNA_db/search.php

**Conclusions:**

In attempt to perform top-down prediction of the human siRNA with minimized off-target hybridization, we developed an efficient algorithm that employs suffix tree based storage of the substrings. Applications of this approach are not limited to optimal siRNA design, but can also be useful for other tasks involving selection of the characteristic strings specific to individual genes. These strings could then be used as siRNA seats, as specific probes for gene expression studies by oligonucleotide-based microarrays, for the design of molecular beacon probes for Real-Time PCR and, generally, any type of PCR primers.

## Background

siRNA-based silencing of the gene expression involves homology-dependent suppression of the cognate mRNA either at the transcriptional or post-transcriptional level [1]. Most important part of this process involves an interaction of target mRNA with string-specific double-strand RNA molecules (siRNAs) of about 21 nt with 3'-overhangs [2]. An annealing of siRNA to unrelated but partially homologous mRNAs produces interference with the silencing process leading to a diminished efficiency [3]. Additionally, mRNAs with partial homology to siRNA molecules may also be degraded to some extent, evoking unwanted physiological effects [4]. In the clinical settings, e.g. when siRNA is applied as an antiviral treatment, it may lead to imbalance of the normal cellular functions that could, in turn, manifests as side effects of the therapy. This phenomenon, called 'off-target' silencing, is known as one of the most serious problems in RNA interference (RNAi) [3,5]. Until major improvement in siRNA design occurs, both the development of siRNA-based therapeutic applications and interpretation of gene function and phenotypes resulting from RNAi experiments will be hindered.

When tested *in vivo*, about 80% of theoretically possible mammalian siRNAs were shown to be not functional or suboptimal [6]. To improve siRNA design, a set of rules for detecting 21-mer target sites was proposed, including a low G+C content, a lack of internal repeats and an A/U-rich 5' end [7]. The importance of certain secondary structures at the siRNA target site [8] and the absence of the short string matches to the 3' areas of other human genes [9] were emphasized. A number of reliable algorithms for the prediction of highly specific and efficient siRNAs have been published [Rev. in [10]]. Nevertheless, minimization of the siRNA off-target effects still needs major improvement.

A typical approach for off-target effects reduction is by the similarity search with the basic local alignment search tool (BLAST) using the organism-specific transcriptome dataset [11]. Use of the BLAST algorithm promptly returns possible secondary targets, but a proportion of the significant alignments may be missed [12]. On the other hand, an exhaustive Smith-Waterman local alignment algorithm [13] returns accurate answers but is so time-consuming that it often requires hardware augmentation [14,15]. Several authors proposed adjustments for mismatch tolerance [12,16] that may lower the effectiveness of siRNA found and, again, are costly to calculate.

One of the possible ways to increase the speed of calculations without losing its specificity and sensitivity is to pre-compute transcriptome-specific sets of gene-specific strings with decreased redundancy ("siRNA seats"). For example, Naito et al. aligned all the human RefSeq and UniGene strings onto the human genomic strings, and retrieved duplicate-free exons and strings over exon-exon junctions and pre-computed gene-specific 19-nt strings with a smaller number of collaborative off-target hits, defined as complete or partial matches of multiple 19 nt substrings [12]. Although representing an important step forward, this approach yields siRNA candidates that may still cause an off-target effect as the stretches of as few as 11-to-15 consecutive nts are enough to produce unwanted silencing [17].

Next improvement has been made by the Comprehensive Redundancy Minimizer (CRM) algorithm that allows one to map all unique short-string strings ("kernels") 9-to-15 nt in size (length "N") within large sets of strings, e.g. an entire transcriptome [18]. CRM algorithm ensures that every predicted siRNA seat of length 21 is comprised of overlapping kernels of length N, where N is between 9 and 17. The CRM-based filtering was tested on two complete transcriptomes, human and murine, and proven efficient using the collection of published sets of siRNAs with known efficacies [19].

Here we suggest an alternative to CRM algorithm that highlights gene-specific siRNA seats with minimized off-target annealing in a cost-efficient way. Our algorithm relies on a search efficient truncated suffix tree data structure. The tree-based organization provides for the saving of the computation time when it comes to both storage and searching for substrings within the gene. The algorithm outputs results in an easily reusable tab-delimited form.

The idea of suffix trees dates back to the concept of a *position tree *introduced in [20]. The construction was greatly simplified by McCreight [21], and also by Ukkonen [22]. Ukkonen provided the first linear-time online construction of suffix trees, now known as Ukkonen's algorithm. This data structure is reminiscent of the binary trees widely used in computer science and in fact suffix trees have been used in the information science literature [23]. In recent years, the concept has found numerous applications in computational biology [24-27]. The data structure we used in this work is closely related to the truncated suffix trees utilized in [25-27]; however, it contains the information about the positions of the substrings in the database and lacks horizontal links which make it more suitable for the siRNA application at hand.

Using the new algorithm, we pre-computed a list of the best siRNA locations within each human gene ("siRNA seats"). The complete list of siRNA locations with minimized off-target hybridization is available at http://web.cos.gmu.edu/~gmanyam/siRNA_db/search.php). These siRNA seats could be used as an input for the traditional "set-of-rules" type of siRNA designing software.

## Main text

### Data structure and problem formulation

Let us now explain in details the type of structure our algorithm for sorting and analyzing the substrings within the entire transcriptome is based on. Each substring of a certain length *n *is stored in a modified *n*-truncated suffix tree with each node having 4 pointers associated with the nucleotides A,C,G or T, so each string is represented by a unique path from the root of the tree to its leaf. The string storing procedure is carried out in the following way.

***Procedure 1 [String Storage Procedure] ****We create a branch from the root of the tree to a vertex in the first level of the tree corresponding to the first character in the string (A,C,G or T)*. *We then create a branch from this vertex to a vertex in the second level corresponding to the second character, and so on until we have reached the n^th ^level of the tree, where n is the total number of characters in the string*. *If a certain branch of the tree already exists, we simply follow that branch to the next level of the tree without the need to create a new branch*.

In the tree, each string is accompanied by certain information, such as its frequency, gene of origin and the position within the gene. String-specific information is stored in each of the leaves, allowing one to avoid unnecessary string comparisons. Furthermore, the storage space is generated on demand, so that no memory is wasted. We call the resulting tree structure with all the information stored in the leaves a **modified n-truncated suffix tree**.

The approach described above is similar to the suffix tree construction used in the Entropic Profiler software introduced in [26]. However, instead of connecting nodes at the same depth within the tree with "side links", we simply store the location of each substring within the database which enables us to save on storage and to "sweep" through the siRNA seat computation as quickly as possible. To explain this distinction, let us formalize some important notions to be used in the description of the proposed algorithm.

***Definition 1***.

*Denote G a set of genes, represented as strings*. *Consider two integers n and N, with n < N*, *with n playing a role of a threshold*. *Let be the set of all substrings of length N in the set *G *(referred to as N-strings)*. *Two strings x and y in U are called **duplicate N-strings **if and only if they belong to different genes and contain at least one pair of substrings of length n (referred to as n-strings) which are equal (i.e*. *there is a substring of x of length which is equal to some substring of y of the same length)*. *A string x is called a **unique N-string **if it is not a duplicate of any other string in U*. *Any unique N-string x in U forms a **siRNA seat***.

**Figure 1:**
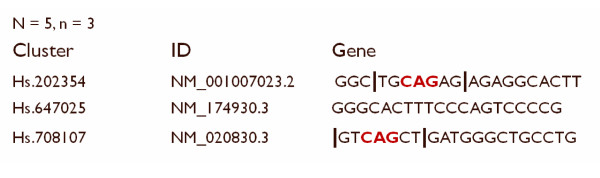
**Illustration of a duplicate string concept**. This example explains how one finds duplicate strings in a transcriptome consisting of 3 genes containing duplicate strings of length N = 5.

Consider an example given in Figure 1. Given *n *= 3 and *N *= 5, the motif CAG (for instance) present in the first and the third gene leads to the following N-strings being duplicates according to the above definition: TGCAG, GCAGA, CAGAG from gene 1 and GTCAG, TCAGC, CAGCT from gene 2 respectively. In the meantime, the *N*-string GAGAG from gene 1 is unique since there is no substring of length 3 in either gene 2 or 3 equal to GAG or AGA.

The problem to be solved by means of these tools can be formally described as follows:

***Problem formulation***.

*Given a gene belonging to G find, from left to right, all maximal substrings of length N that are unique according to the threshold n*. *Generate a database consisting of these unique strings (siRNA seats) collected from all genes in G*.

Notice that all seats are of the same length *N *unless they happen to be on the boundary of the original gene, in which case they can be as short as *n *characters. The minimal length of a siRNA seat is *n*.

Algorithm description

Given a database of genes and a fixed positive integer *n*, the algorithm stores all *n-*substrings for the entire collection of genes. Additionally, for any *n*-string, we store the location of each occurrence of this *n*-string under the same index of the tree. Sorting the strings facilitates downstream analysis on the data. More precisely, the tree-sort algorithm takes as input a database of genes, comprised of the nucleotides A, C, G, and T. To store all possible *n-*strings, we need a full tree with *n+*1 levels (counting the root), labelled 0 through *n*. Since there are four types of nucleotides, the *k*^th ^level has at most 4*^k^*vertices. Each vertex in the *k*^th ^level has branches to four vertices in the (*k+*1)^th ^level. In order to locate siRNA seats once the suffix tree has been built, we first print the list of all unique substrings in the list UNIQUE_n, which also contains the associated gene symbol and the location of the substring within that gene. After the unique strings have been identified in this fashion, the siRNA "seats" can be generated on-the-fly without re-reading the transcriptome. Indeed, for each unique subsequence specified in the list UNIQUE_n, we need to look at the *N *- *n *characters succeeding it in the original gene. If not all of the *N *- *n *characters are available due to the proximity of the gene boundary, only available characters are taken into consideration. All strings of length *N *in the resulting set are checked for uniqueness. Each unique string found this way forms a new siRNA seat. Algorithm 1 formalizes the steps described above.

***Algorithm 1***. ***Suffix tree-based calculation of siRNA seats of length N with threshold n***

***Input: G ****- the set of all genes in the database, a threshold value n and the siRNA length N*.

***Output: ****siRNA seats of length N with threshold value n*.

*Put i = *1. *Initialize the vector *[0, ..., 0].

***While ****(the set of remaining n-strings in G is non-empty)*

*Read an n-string from and denote it s_i_*. *Store s_i_in the suffix tree according to Procedure 1*. *Store the corresponding gene ID and the location of the string within that gene in the leaf*. *If the substring s_i _already exists in the tree and belongs to a different gene, set counter_i _= *1 *to reflect the fact it is a duplicate*.

*i *= *i *+ 1

*End while*

*Identify leaves with counter_i_*= 0 *(unique n-strings) and save their location in UNIQUE_n*.

*Let n_unique_be the total number of n-strings in UNIQUE_n*.

***For ****(j from *1 *to n_unique_)*

*For the j-th n-string in the list UNIQUE_n, find a (larger) N-string containing it by scanning all available N - n characters to the right of it in the same gene (found by gene ID)*. *Mark the resulting N-string as unique if all of its n-substrings exist in the list UNIQUE_n (Definition 1)*.

*End for*

***Return ****all unique N-strings - these represent all siRNA seats found in G*.

**Figure 2:**
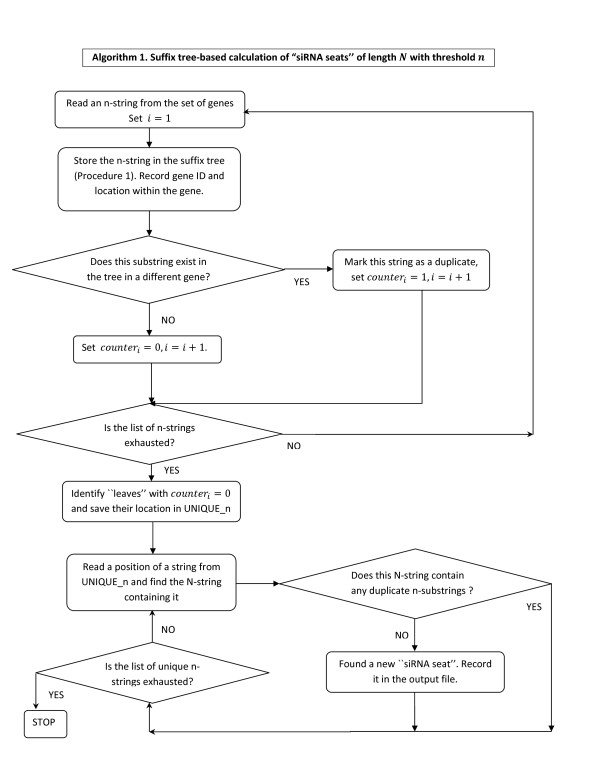
**Algorithm 1 flowchart**. Algorithm 1 performs suffix tree-based calculation of "siRNA seats'' of length *N *with threshold *n*.

**Figure 3:**
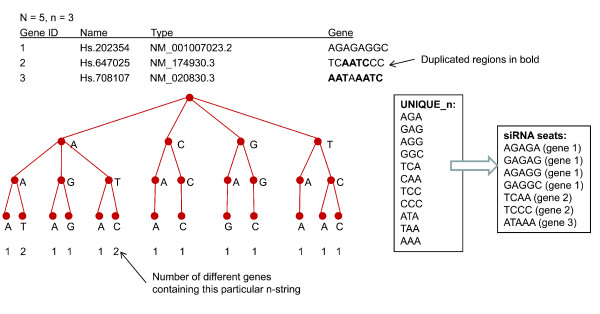
**Generation of the storage of all substrings**. This example illustrates the steps of Algorithm 1 for the input consisting of 3 genes AGAGAGGC, TCAATCCC and AATAAATC. All of the corresponding n-strings are identified with the number of occurrences stored in the leaf. The list of unique n-strings is provided, and the "siRNA seats" resulting from this computation are specified.

The flowchart description of this algorithm is given in Figure 2. In Figure 3 we provide an illustration of the steps the algorithm performs to store each of the input nucleotide strings and to identify the corresponding siRNA seats for some sample data. In this simple example with only 3 genes present, the suffix tree generated at Step 3 has 13 leaves with 11 of them correspond to unique n-strings recorded in the list UNIQUE_n. The output is given in a form of 7 unique N-strings which we refer to as ''siRNA seats''.

It is worth noting that the tree construction utilized by Algorithm 1 allows for quick modification of the results in case new genes are added to the database, with no need to re-create the suffix tree. The new gene information in the form of a collection of -strings will be recorded in the tree based on Procedure 1 and which will result in incremental changes to the list UNIQUE_n. Any possible duplicates arising from this change will be immediately detected when performing Steps 4-5 of the Algorithm and will allow for fast recalculation of the siRNA seats.

### Memory requirements

The new algorithm requires the following four main types of memory: character arrays to store the gene strings, gene structures containing pointers to the each field in the gene strings, storage structures to track the location of each *n*-string, and the tree structures to sort the *n *-strings. Suppose our database of genes has *G *genes with average length *L*. Then the memory for the gene structures is *G*Δ*L*. Each gene structure contains a pointer to the gene cluster, a pointer to the gene ID, a pointer to the actual gene string, and an integer containing the length of the string. Using 32-bit pointers, each gene structure requires 16 bytes of memory, and we need *G *gene structures. Thus the total memory for the gene structures is 16*G *bytes. We need a storage structure for each *N*-string in the database. That number is equal to *G*(Δ*L *+ 1- *n*). Since *n *≪ Δ*L*, we use *GΔL*. The storage structure contains a pointer to the associated gene, an integer specifying the location of the *N*-string in the gene, and a pointer to another storage structure containing the previous occurrence of this exact *N*-length string. So the total memory for the storage structures is approximately 12*G*Δ*L *bytes.

For the character arrays, gene structures, and storage structures, we need a combined *G *(13 Δ*L *+ 16) bytes of memory, which amounts to 700 MB of RAM. The tree structures contain four pointers to the next level of the tree, a pointer to a storage structure, and an integer to count the occurrences of the associated *n*-string. Thus, each tree structure requires 24 bytes of memory. The exact number of tree structures necessary depends on *n *and the types of *n*-strings in the database. Table I shows how many branches were used for each value of *n*. The right column is the tree memory plus 700 MB from the other three main components. For larger values of *n*, the full tree requires a significant amount of storage space. However, as the algorithm generates storage space on demand, we only need to create branches and vertices in the tree to store the *n*-strings that are actually present in the transcriptome. Interestingly, we noted that as *n *gets larger, the ratio of present *n*-strings to all possible *n*-strings decreases. In fact, for *n *= 16, we utilized only 3% of the theoretically complete exhaustive tree to store all 16-length strings present in human transcriptome, as demonstrated in Table 1.

**Table 1:** The size and fill-in of the tree needed to store the initial dataset and the memory consumption

N	Branches in Full Tree	Branches Used	% Branches Used	Full Tree Memory	Actual Tree Memory	Total Memory
**9**	349525	349519	99.998%	8 MB	8 MB	708 MB
**10**	1398101	1395271	99.798%	32 MB	31.94 MB	731.94 MB
**11**	5592405	5366925	95.968%	128 MB	122.84 MB	822.84 MB
**12**	22369621	17849905	79.795%	512 MB	408.55 MB	1.08 GB
**13**	89478485	45717780	51.094%	2 GB	1.02 GB	1.70 GB
**14**	357913941	88264307	24.661%	8 GB	1.97 GB	2.65 GB
**15**	1431655765	138965433	9.707%	32 GB	3.11 GB	3.79 GB
**16**	5726623061	192967338	3.370%	128 GB	4.31 GB	5.00 GB

### Implementation and testing

**Figure 4:**
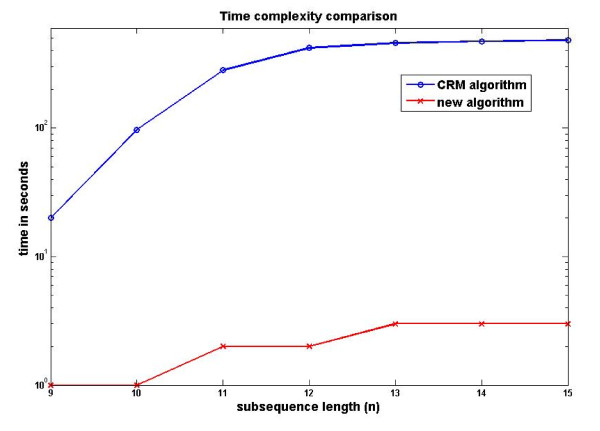
**Algorithm efficiency comparison**. Time taken to retrieve all unique strings of length *n *for the new algorithm (crosses) and the previously suggested CRM algorithm (circles) on a logarithmic scale.

The new algorithm was implemented in C and compared to the CRM algorithm described in [18]. Both codes were tested on a 640-core SGI Altix cluster. The comparative review of the performance is presented in Figure 4. The new algorithm was able to retrieve the list of unique substrings of length *n*, where 9 <*n *< 15 on average 100 times faster than the CRM. The time required for the generation of CRM input, the string pre-sorting time, is not reflected in the efficiency comparison, while the new algorithm has a built-in sorting routine whose execution time was taken into account. The advantage of the new method would be even more visible had the time to pre-sort the list been counted as part of the CRM execution time too.

### Practical application

To demonstrate the utility of the novel algorithm, we applied it to parse a non-redundant set of human transcripts onto the 11 to 17 nucleotides substring and extract the sets of siRNA seats comprised of substrings with given length (Figure 3).

Human mRNA strings were extracted from the NCBI Unigene dataset (build #219). For each gene, the longest reference mRNA string with NM identified was extracted and further processed using the new algorithm.

Predicted siRNA seats were placed in a mySQL database. To provide an access to the siRNA seats stored in this database, a web interface was built using PHP. User-friendly interface of the database allows the search with the HUGO approved Gene Symbol, NCBI Entrez Gene ID, Genbank Acession or Unigene cluster ID for siRNA seats comprised of unique oligonucleotides with selected length. As an output, siRNA seat database lists all seats with lengths equal or larger than 19 nucleotides, with their relative positions within respective mRNA string. For the convenience of the user, each siRNA seat search also returns the string of the mRNA template used for the tree parsing and some general information about the gene of query including its exon/intron structure. We envision that users may consult siRNA seats database before embarking on siRNA design as gene-specific lists of siRNA seats with minimized off-target hybridization may be used as input for any conventional siRNA designing software instead of the entire string corresponding to the gene of interest. The searchable database of all possible human siRNA seats is available at http://web.cos.gmu.edu/~gmanyam/siRNA_db/search.php.

## Discussion

String-specific small interfering RNAs (siRNAs) could be used both as therapeutic molecules and as a new instrument for a drug target discovery. Cellular and animal models already demonstrated the potential of siRNA-based treatments for cancer, viral infections and inflammatory diseases. However, the development of siRNA based therapeutics is hampered by 'off-target' silencing effects that have to be minimized in order to diminish the possibility of the side effects.

One relatively straightforward approach to 'off-target' minimization is to design siRNA molecules for pairing up with unique locations within mRNA targets. The traditional "bottom-up" approach to siRNA design implies an exclusion of any possible short string matches using BLAST or Smith-Waterman algorithm. On the other hand, one may employ "top-down" approach by using a pre-computed set of least redundant locations within the entire transcriptome (siRNA seats) as input for the traditional siRNA designing software. "Top down" approach requires less siRNA designing skills form a novice researcher and limits the set of gene-specific candidate siRNAs to a smaller number of molecules in need of experimental verification. However, the transcriptome-wide extraction of the least redundant substrings is not a trivial task. The first algorithm of this kind, CRM [18] that successfully completed the task, was far from efficient.

Here we propose a substantially more efficient algorithm that employs tree based storage of the substrings, which is the first application of this mathematical concept in this context. The approach developed here is not limited to optimal siRNA design, but can also be useful for other tasks, such as selecting characteristic strings specific to individual genes in certain organisms. These strings could then be used as siRNA seats, as specific probes for gene expression studies by oligonucleotide-based microarrays, for the design of molecular beacon probes for Real-Time PCR and, generally, any type of PCR primers.

Another important advantage of the new algorithm over CRM is that the storage structure created by the new algorithm automatically records the frequency for each substring. Therefore, this suffix tree based approach can be easily utilized to perform other types of transcriptome analysis, including a search for unique substrings and absent substrings, analysis of distributions of the substrings associated with various biological features, e.g. promoters, 3' untranslated regions and open reading frames. This further analysis is the subject of an ongoing study.

Among the limitations of the proposed algorithm are 1) the necessity of the periodical re-analysis of the available siRNA seats within transcriptome in order to incorporate newly discovered functional RNA transcripts; and 2) inevitable miss of the imperfect siRNA seats that might couple with respective siRNA and act as "seed rule" violation but nonetheless efficient miRNAs instead. Latter possibility needs to be studied experimentally by systematic analysis of the rejected siRNA seats using miRNA recognizing algorithms, and is included in the plan for the future development.

## Conclusions

Here we present a new efficient suffix tree-based algorithm that delivers a comprehensive and systematic analysis of substrings within an arbitrary set of biological strings. The proposed algorithm may help to find biologically significant features within large gene databases. In this paper, we described an application of this algorithm to exhaustive search for the "siRNA seats" in entire human transcriptome. Resulting database of siRNA seats is available at http://web.cos.gmu.edu/~gmanyam/siRNA_db/search.php.

## Competing interests

The authors declare that they have no competing interests.

## Authors' contributions

AB conceived the ideas of the top-down screening for siRNA seats and the necessity of the high-throughput analysis of the string substrings; she also contributed to the manuscript writing. JB developed and implemented the suffix-tree based algorithm under the guidance of ME. GM developed mySQL database and the web site for siRNA search. ME advised on the development of the new algorithm, its analysis and implementation, and contributed to the manuscript writing. All authors read and approved the final manuscript.
